# Decision Criteria for Large Vessel Occlusion Using Transcranial Doppler Waveform Morphology

**DOI:** 10.3389/fneur.2018.00847

**Published:** 2018-10-17

**Authors:** Samuel G. Thorpe, Corey M. Thibeault, Nicolas Canac, Seth J. Wilk, Thomas Devlin, Robert B. Hamilton

**Affiliations:** ^1^Neural Analytics, Inc., Los Angeles, CA, United States; ^2^Erlanger Medical Center, Chattanooga, TN, United States

**Keywords:** transcranial doppler, ultrasound, ischemic stroke, large vessel occlusion, decision tree, diagnostic biomarker

## Abstract

**Background:** The current lack of effective tools for prehospital identification of Large Vessel Occlusion (LVO) represents a significant barrier to efficient triage of stroke patients and detriment to treatment efficacy. The validation of objective Transcranial Doppler (TCD) metrics for LVO detection could provide first responders with requisite tools for informing stroke transfer decisions, dramatically improving patient care.

**Objective:** To compare the diagnostic efficacy of two such candidate metrics: Velocity Asymmetry Index (VAI), which quantifies disparity of blood flow velocity across the cerebral hemispheres, and Velocity Curvature Index (VCI), a recently proposed TCD morphological biomarker. Additionally, we investigate a simple decision tree combining both metrics.

**Methods:** We retrospectively compare accuracy/sensitivity/specificity (ACC/SEN/SPE) of each method (relative to standard CT-Angiography) in detecting LVO in a population of 66 subjects presenting with stroke symptoms (33 with CTA-confirmed LVO), enrolled consecutively at Erlanger Southeast Regional Stroke Center in Chattanooga, TN.

**Results:** Individual VCI and VAI metrics demonstrated robust performance, with area under receiver operating characteristic curve (ROC-AUC) of 94% and 88%, respectively. Additionally, leave-one-out cross-validation at optimal identified thresholds resulted in 88% ACC (88% SEN) for VCI, vs. 79% ACC (76% SEN) for VAI. When combined, the resultant decision tree achieved 91% ACC (94% SEN).

**Discussion:** We conclude VCI to be superior to VAI for LVO detection, and provide evidence that simple decision criteria incorporating both metrics may further optimize.

**Performance:** Our results suggest that machine-learning approaches to TCD morphological analysis may soon enable robust prehospital LVO identification.

**Registration:** Was not required for this feasibility study.

## Introduction

Acute Ischemic Stroke (AIS) is the leading cause of long-term disability in the United States, and fifth leading cause of death (1). Current treatment for AIS includes the use of intravenous tissue Plasminogen Activator, and endovascular mechanical thrombectomy with a clot extraction or aspiration device. Although these therapies provide effective treatment options for Large Vessel Occlusion (LVO), their use is still limited by short time windows from symptom onset during which they are optimally effective (2–4). Indeed, only a small fraction of candidate patients who could ultimately benefit from endovascular treatment currently receive it (5). Early LVO identification is key to enabling rapid triage and transfer to comprehensive stroke centers, thus facilitating access to appropriate care. Computed Tomography Angiography (CTA) is the current gold standard for stroke diagnosis, but is limited to in-hospital use, or a low number of prohibitively expensive mobile stroke ambulances. Unfortunately, current prehospital stroke assessment scales lack reliability due to training requirements and low inherent accuracies (6, 7); causing delays in triage, transfer, and treatment.

Transcranial Doppler (TCD) ultrasound is a reliable diagnostic tool for assessing the presence and severity of LVO (8–11), which has the additional advantages of being non-invasive, inexpensive, and portable. Because it directly measures Cerebral Blood Flow Velocity (CBFV), TCD is a strong candidate technology for prehospital diagnosis and assessment of LVO. Indeed, bedside TCD examinations to detect stenosed and/or occluded intracranial vessels are routinely conducted as standard of care at many comprehensive stroke centers (12). Numerous studies have been published comparing TCD diagnosis of arterial LVO with CTA imaging; reporting sensitivity (SEN) and specificity (SPE) ranging between 79 and 98% depending on occlusion location (13–17). A limiting factor of these studies is the TCD operator's ability to locate and interpret the CBFV waveform. Such challenges have contributed to TCD being critically underutilized for stroke assessment.

For stroke diagnosis, specialized training is required to inspect flow velocity and morphology across multiple vessels and depths in both cerebral hemispheres. One of the most cited papers for stroke diagnosis using TCD was published by Demchuk et al. (10), which instructs the operator to categorize waveforms according to evidence of stroke-related pathology; namely dampened, blunted, minimal, or absent signal. A number of additional TCD exam methodologies with different criterion for LVO assessment have been published (15, 17). Typically, CBFV and power M-mode (PMD) waveforms are obtained for flow through the Middle, Anterior, and Posterior Cerebral Arteries (MCA, ACA, and PCA) in each cerebral hemisphere, as well as the Internal Carotid Arteries (ICA). Heuristic assessments are then made based on numerous features, including relative velocities, collateral flow, PMD resistance signatures, and the presence of pathological waveform morphologies.

Assessment of these categories relies heavily on qualitative interpretation by specialists which cannot be replicated by less formally trained personnel. The challenge of moving LVO detection to the prehospital setting thereby obviates the need for objective metrics by which first responders might reliably evaluate TCD signals. An intuitive first candidate for such a metric is CBFV asymmetry, as it is already well established that velocity disparity, both between homologous vessels in opposite hemispheres (10, 18) as well as adjacent vessels in an occluded hemisphere (10, 15), can be indicative of vascular occlusion. One promising metric for LVO detection based on velocity disparity was published by (15); showing area under the Receiver Operating Characteristic curve (ROC-AUC) of 92.6%. However, their metric also relied on PMD resistance signatures as a predictive feature, which were not objectively computed, and was limited in application to occlusions of the MCA.

However intuitive, assessment of velocity asymmetry also comes with the inherent concern that velocity estimates in adjacent vessels and opposite hemispheres can be greatly impacted by anatomical variability (incident angle of the vessel and ultrasound beam), as well as by intrarater measurement inconsistency (19). Moreover, reliance on mean velocity disparity inherently discards the morphological information currently utilized in routine stroke assessment protocols. Such assessments incorporate morphological information explicitly, but in a subjective manner which requires expert interpretation. However, a number of recent studies have observed morphological changes associated with various medical conditions which are both objectively quantifiable (20–22), and independent of significant changes in mean velocity (23). Pulsatility Index is an example of a well known and widely clinically utilized morphological TCD variable (24); one which evidence suggests is not useful for detecting LVO (15).

Toward the aim of quantifying TCD waveform morphology for the purpose of LVO identification, we have recently proposed a diagnostic biomarker termed Velocity Curvature Index (VCI) (25, 26). Mathematically, it is a straightforward extension of the concept of graph curvature; one which is sensitive to the morphological structure of the pathological waveforms first described by Demchuk et al. (10). In this work we retrospectively compare the diagnostic utility of VCI to that of a standard Velocity Asymmetry Index (VAI) for the detection of LVO in a clinical subject population collected in-hospital. Additionally, we evaluate a simple decision tree classifier designed to incorporate complimentary information from both metrics. Decision trees are a commonly used diagnostic methodology in several areas of medicine (27, 28), which have previously been used with TCD variables to screen for cervical vascular injury (29). To these ends, we employ leave-one-out cross validation and subsequent sensitivity analysis to assess performance as diagnostic thresholds are weighted toward detection of true positives. Our goal is the validation of TCD-based decision criteria which are objective, intuitive, and easily communicated; allowing physicians and first responders alike a common language for LVO assessment.

## Materials and methods

### Subject examination and imaging

We acquired TCD waveforms from two clinical populations enrolled consecutively at Erlanger Health System's Southeast Regional Stroke Center in Chattanooga, TN, from October 2016 through September 2017. The LVO group was comprised of patients with CTA-confirmed occlusion of the M1/M2 branches of the MCA and/or ICA (proximal extracranial or terminal intracranial segments); these occlusion locations being selected since they are the large cerebral arteries most amenable to neurovascular intervention. The In-Hospital Control group (IHC) consisted of patients who arrived at the hospital presenting with stroke symptoms, but were later confirmed negative for LVO by imaging. Patients in both groups received TCD examinations in addition to standard care (patient history, monitoring, pharmaceuticals, and CT/A/perfusion imaging). Patients were eligible for enrollment if a complete TCD exam was acquired within 4 h of CTA, and none of the following exclusions applied: (1) Head CT findings consistent with acute primary intracranial hemorrhage, (2) Hemodynamically unstable patients requiring pharmacological support for hypotension; (3) Subjects who underwent partial or full craniotomy; (4) Additional intracranial pathologies present (tumor, hydrocephalus, etc.); (5) Anticipated insufficient time to acquire a complete set of scan as described by the protocol; (6) Significant hemodynamic pharmacological agent (cocaine, amphetamine, etc.); (7) Subjects who are under arrest for a felony.

CTA examinations were performed using a GE Lightspeed VCT 64-section multidetector scanner (GE Healthcare, Milwaukee, WI) with a slice thickness of 0.625 mm, and bolus injection of 70–150 mL of Omnipaque 350 (GE Healthcare, Milwaukee, WI) contrast material (4.0 mL/s). CTA images were reformatted in the coronal and sagittal plane, and 10-mm maximum intensity projection reconstructions were rendered and sent to PACS for review. Occlusion location was determined by the radiologist on call, who was blinded to any results of the TCD examination.

**Figure 1:**
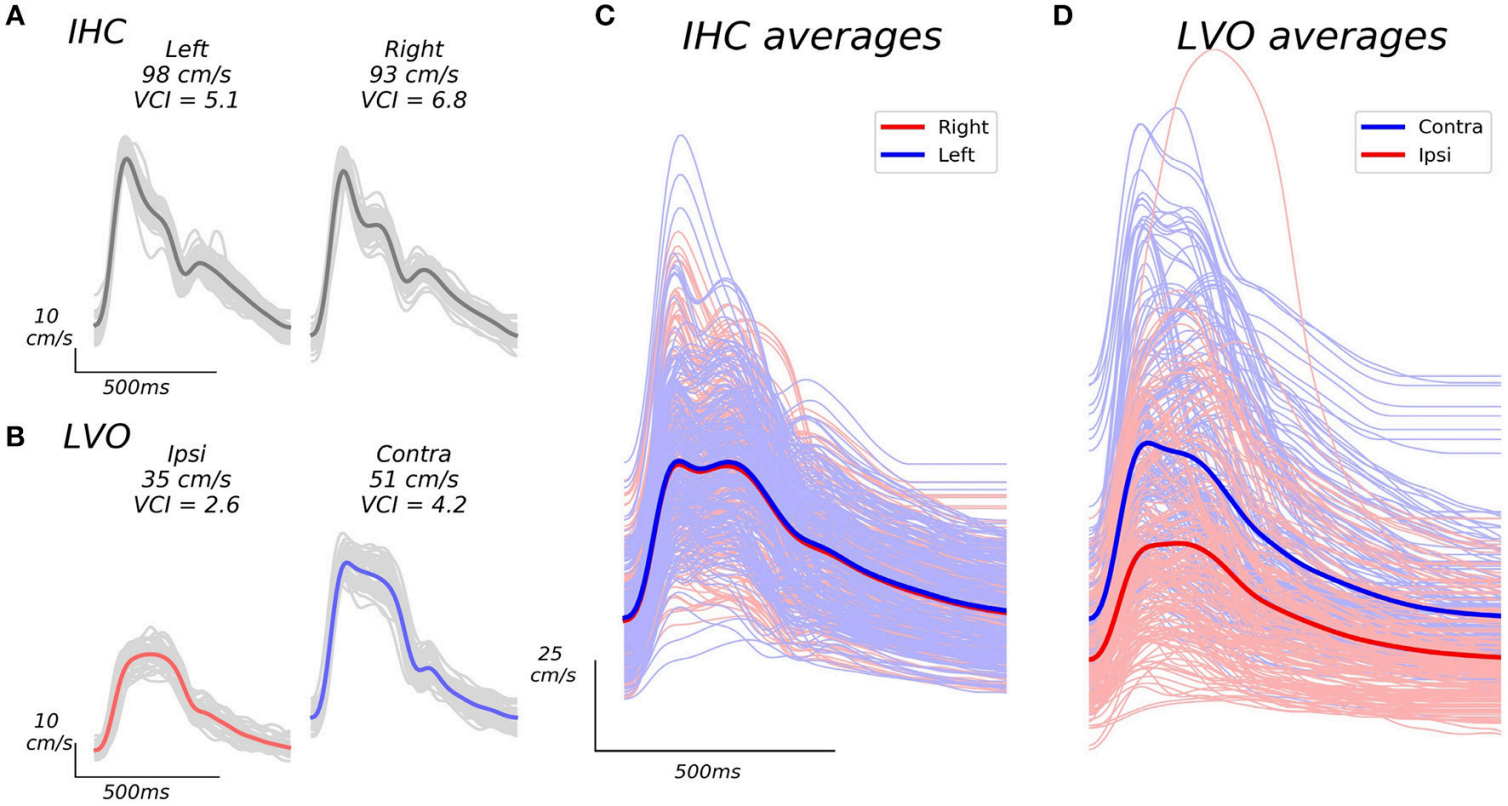
Example beat waveforms from IHC **(A)**, and LVO **(B)** groups, along with associated group averages **(C,D)**. The IHC example subject recordings shown in A display high VCI in both hemispheres, and relatively symmetric bilateral velocities (VAI = 0.95). The LVO subject recordings depicted in B show decreased VCI which is especially pronounced in the ipsilateral hemisphere (same side as occlusion as confirmed by CTA), as well as less symmetric velocity (VAI = 0.69). Light gray traces in **(A)**, and **(B)** depict the individual beat ensemble (recorded over 30 s) from which each average beat waveform is derived. Grand averages across average beat waveforms for all subject recordings are shown in **(C,D)**. The IHC grand averages shown in C exhibit similar expected velocity across left and right hemispheres, whereas LVO grand averages **(D)** exhibit pronounced inter-hemisphere disparity relative to occlusion location, with markedly reduced curvature especially noticeable in ipsilateral recordings.

Complete TCD examinations included (at minimum) one pair of left/right MCA scans at depths between 45 and 60 mm, each containing 15 or more individual beat waveforms (see Figure 1). Subjects for whom complete examinations were not obtained were counted as missing/indeterminate data and excluded from analysis. Data was acquired during available intervals between patient testing/treatment, and in no way impacted patient care. The TCD technician was often present during initial evaluation of the subject, and so was not entirely blinded to all clinical information or imaging results. Sample size was not predetermined for this feasibility study, being established pragmatically as the maximum number of subjects attainable in the allotted time frame. Experiment protocols were approved by University of Tennessee College of Medicine Institutional Review Board (ID: 16-097). Reporting in this manuscript is structured in accordance with the Standards for Reporting of Diagnostic Accuracy Studies [(30); see Appendix for detailed criteria checklist].

### Waveform processing and feature extraction

#### Recording

TCD scans were acquired by a trained technician using 2 MHz hand-held ultrasound probes. CBFV signals associated with the left/right MCA were identified via insonation through the transtemporal window. CBFV envelopes were digitally sampled at 125 Hz and recorded throughout the entire exam. Once the CBFV signal was identified and optimized at a specific depth, waveform recordings were then made in 30-s intervals. The technician was instructed to obtain recordings for as many depths as possible between 45 and 60 mm in both the left/right cerebral hemispheres. Start times for each interval were marked by the technician using a custom event remote, which prompted a 30 s countdown to a corresponding stop event. TCD envelopes and event information were aligned using custom software (Python 2.7; Kivy 1.9) running on Windows 10.

#### Processing

Average beat waveforms from each recorded depth interval were extracted using a combination automated beat identification algorithm with manual checking/editing. In this procedure, individual beats within each interval were first identified automatically using an internally developed beat extraction tool, and displayed to the user for manual confirmation/editing. Detected beats which lacked clear pulsatile structure and/or deviated anomalously from the group average (usually due to probe displacement during recording), were excluded. The remaining beats were then aligned and averaged, resulting in a single representative beat waveform for each recorded depth interval (see examples in Figure 1).

Since Doppler velocities scale with the cosine of the incident angle between the ultrasound beam and underlying blood flow (31), TCD waveforms for a given vessel with the highest measured velocities are assumed to most accurately reflect reality. In line with this reasoning, for each subject we selected a single bilateral (left/right) pair of average beat waveforms for analysis consisting of those with maximal mean velocity across all recorded depths for each hemisphere.

#### VCI

Curvature is a well-defined mathematical property of space curves which quantifies the degree to which a curve deviates from being “straight” at a given point. VCI is an application of the curvature metric specific to TCD which quantifies the degree to which a beat is blunted and/or dampened. Since curvature is a nonlinear function sensitive to small inflections associated with high frequency noise, we first smooth the average beat waveform via convolution with the Hanning window (9 ms). Moreover, we elect to consider only curvature associated with the beat systolic complex, where the signal-to-noise ratio is presumably greatest. The systolic complex, or “beat canopy,” comprises the proportion of the beat with the highest velocities and richest morphological structure.

**Figure 2:**
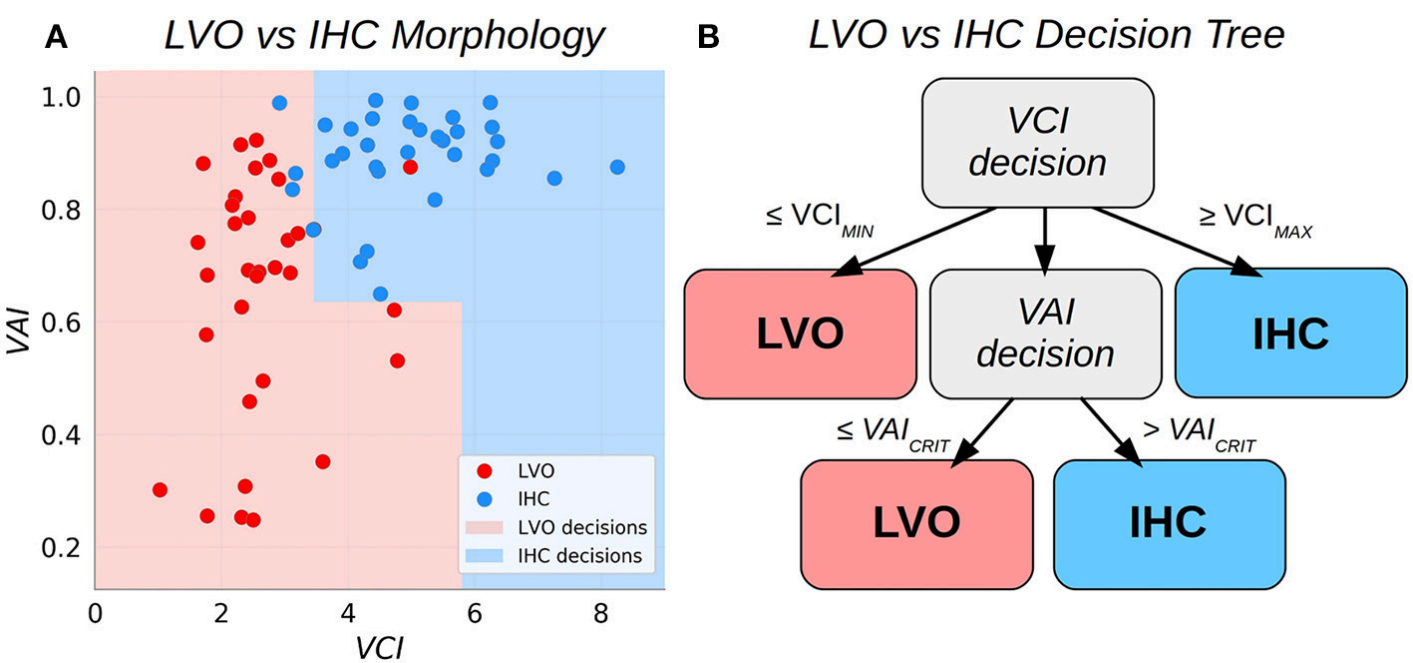
Scatter plots relating the Velocity Asymmetry Index and Velocity Curvature Index **(A)**, along with a decision tree partitioning the two-dimensional feature space **(B)**.

To compute VCI for a given TCD beat waveform, curvature is first computed for each time point (*t*_*i*_) of the smoothed beat (denoted *x(t*_*i*_*)* below) via the discretized equation for graph curvature (equation 1) expressed in terms of finite differences. Δ and δ^2^ in equation 1 denote the first order backward (equation 2) and second order central (equation 3) finite difference equations. VCI, defined by equation 5, is computed as the sum of curvature taken over all individual time points comprising the beat canopy (*C*). The beat canopy is defined in equation 4 as the set of time points wherein velocity exceeds one quarter of its total diastolic-systolic range (*t*_*d*_, and *t*_*s*_ denoting the time points corresponding to diastolic minimum and systolic max, respectively). Since the hypothesized effect of occlusion on the TCD waveform is to lower VCI in the occluded vessel, when assessing a bilateral pair of waveforms we take VCI as the minimum computed for each member of the pair. VCI is a positive metric which, in principle, has no upper bound, but is clearly bounded in practice (see Figure 2A).

(1) $$\begin{array}{l} k\left(t_{i}\right)=\frac{\left|\delta^{2}\left[x\right]\left(t_{i}\right)\right|}{{\left(1+{\left(\Delta \left[x\right]\left(t_{i}\right)\right)}^{2}\right)}^{\frac{3}{2}}} \end{array}$$

(2) $$\begin{array}{l} \Delta \left[x\right]\left(t_{i}\right)=x\left(t_{i}\right)-x\left(t_{i-1}\right) \end{array}$$

(3) $$\begin{array}{l} \delta^{2}\left[x\right]\left(t_{i}\right)=x\left(t_{i+1}\right)-2x\left(t_{i}\right)+x\left(t_{i-1}\right) \end{array}$$

(4) $$\begin{array}{l} C=\left\{i:x\left(t_{i}\right)\geq x\left(t_{d}\right)+\frac{x\left(t_{s}\right)-x\left(t_{d}\right)}{4}\right\} \end{array}$$

(5) $$\begin{array}{l} VCI=\sum_{i\in C}k\left(t_{i}\right) \end{array}$$

#### VAI

Velocity Asymmetry Index is a metric which quantifies the degree to which average CBFV observed for a vessel in a given cerebral hemisphere differ from that observed in the corresponding vessel in the opposite hemisphere (see LVO example in Figure 1). The hypothesis that CBFV in an occluded vessel may be lower than that of the corresponding unaffected hemisphere is intuitive, but also supported by previous work (18). For a bilateral pair of left/right average beat waveforms, denoted *x*_*L*_*(t)* (with *N*_*L*_ total time points), and *x*_*R*_*(t)* (with *N*_*R*_ time points) in equations 6 and 7, respectively, VAI (defined in equation 8) is computed as the minimum average velocity across hemispheres divided by the corresponding maximum. By definition, VAI is a positive definite metric bounded on the closed interval [0, 1].

(6) $$\begin{array}{l} \mu_{L}=\frac{1}{N_{L}}\sum_{i=1}^{N_{L}}x_{L}\left(t_{i}\right) \end{array}$$

(7) $$\begin{array}{l} \mu_{R}=\frac{1}{N_{R}}\sum_{i=1}^{N_{R}}x_{R}\left(t_{i}\right) \end{array}$$

(8) $$\begin{array}{l} VAI=\frac{\min\left(\left\{\mu_{L},\mu_{R}\right\}\right)}{\max\left(\left\{\mu_{L},\mu_{R}\right\}\right)} \end{array}$$

### Feature statistical analysis

For both VCI and VAI, resultant group samples were not normally distributed. Accordingly, we tested significance of observed differences in group distributions for each feature using the Mann-Whitney U test statistic. The U-statistic is directly proportional to the common language effect size (by a factor of the product of the group sample sizes under comparison), which is equivalent to the area under the Receiver Operating Characteristic curve (ROC-AUC). Additionally, we computed ROC curves detailing separability of subject group distributions (LVO vs. IHC) for each feature. Specifically, the ROC curves give True Positive Rate (TPR) as a function of False Positive Rate (FPR) for each possible feature threshold. To quantify binary classification performance, we computed SEN, SPE, and accuracy (ACC) at the thresholds which maximized Youden's J-statistic (32):

(9) $$\begin{array}{l} J=TPR-FPR \end{array}$$

Additionally, we bootstrapped 95% confidence intervals (CI) on group means for each feature; accomplished by iteratively resampling each group distribution with replacement (10,000 iterations), and each time taking the mean (CI given by the 2.5th and 97.5th percentile of the resultant empirical distribution) (33). Statistical tests and ROC curves were computed using standard python libraries; SciPy version 1.0, and Scikit-learn version 0.19.1 (34), respectively.

### LVO classification

#### Decision tree classifier

We sought to combine VCI and VAI into a single binary classifier with simple and intuitive decision criteria. The approach adopted here is to augment the bilateral VCI assessment such that subject pairs with VCI less than some low critical threshold are classified as LVO, whereas pairs exceeding some high critical threshold are classified as IHC. Pairs observed to fall between these thresholds are deemed uncertain and decided then based on VAI. This procedure effectively partitions the 2D decision space into two subspaces with piece-wise linear boundaries (see Figure 2). The procedure for fitting the thresholds based on a given set of training data were as follows. First, the low VCI threshold (*VCI*_*MIN*_) was fit using all the training data. Subjects with paired VCI below the threshold were predicted as LVO, and set aside. Next, the high VCI threshold (*VCI*_*MAX*_) was fit from the remaining data. Subjects with supra-threshold VCI were predicted as IHC, and set aside. Finally, the remaining data was used to fit the VAI threshold (*VAI*_*CRIT*_), with sub/supra-threshold subjects predicted as LVO/IHC, respectively. Specifically, each of the three thresholds (*VCI*_*MIN*_, *VCI*_*MAX*_, and *VAI*_*CRIT*_) were fit as the threshold which maximized Youden's J statistic for the data applicable to each decision (see also the sensitivity weighted J-statistic used to determine thresholds for sensitivity analysis in section Sensitivity Analysis).

#### Model cross-validation

For both individual diagnostic metrics (VAI and VCI), as well as the decision tree model, leave-one-out cross-validation was performed to assess generalization of classification performance near decision boundaries. In this iterative procedure, a single subject is removed from the pooled data, and the predictive model is derived via training (i.e., threshold optimization) with the remaining subjects. The excluded subject's data is then predicted using the trained model, and this procedure is repeated for each subject to obtain a complete set of cross-validated predictions from which to assess binary classification performance metrics (SEN/SPE/ACC).

#### Sensitivity analysis

For many clinical problems, including LVO detection, diagnostic net benefit is optimized by increased detection of true positives at the cost of missing true negatives (i.e., SEN is prioritized over SPE). However, poor diagnostics for which SEN is maximized often have no clinical value, as SPE may plummet and overall ACC approaches chance. In order to assess how performance characteristics of our classifiers changed when priority is weighted toward increased sensitivity, we performed a sensitivity analysis wherein we iterated cross-validation of each model, each time incrementing classification thresholds away from the starting point of Youden's maximal J, toward increasing sensitivity. The procedure can be conceptualized as simply adjusting the thresholds up along the associated ROC curves toward increased true positive rate. Mathematically, this was accomplished by introducing a parameter (α) to modify the formula for Youden's J statistic as given in formula 9, and choosing thresholds to maximize the resultant index (*J*_α_). Classifier performance was assessed by cross-validating each model with α ranging from 0.5 (threshold equivalent to Youden's maximal J) to 1 (maximal sensitivity) in steps of 0.01.

(10) $$J_{\alpha }=\text{α}TPR-\left(1-\alpha \right)FPR$$

## Results

### Subject demographics

The current analyses included 33 LVO subjects (16 female), and 33 IHC subjects (13 female), with average ages of 66.9 years (SD = 15.7), and 56.4 years (SD = 16.3), respectively. A total of 156 subject screenings were attempted at Erlanger Medical Center, of which 68 were excluded due to screening failures (time required to complete exam, subject compliance, etc.). Of subjects with sufficient initial screenings, 50 and 38 were initially enrolled in the LVO and IHC groups, respectively. Of the LVO subjects, 3 were discontinued (subject either expressed desire to discontinue, or was transferred or died before enrollment could be completed). An additional 14 LVO and 5 IHC subjects were subsequently excluded due to the presence of disqualifying criteria unknown at the time of enrollment. In the LVO group, there were 20 subjects with M1 occlusions, 3 with M2 occlusions, and 8 with ICA occlusions. An additional subject had dual occlusions of both the M1 and ICA (same hemisphere), and another additional subject had bilateral occlusions of both ICA, in addition to an M2 occlusion. TCD exams were performed an average of 33 min (SD = 20) post-CTA for IHC subjects compared to 43 min (SD = 44) post-CTA for LVO subjects (difference not significant between groups; *t* = −1.15, *p* = 0.26). At time of admittance, LVO subjects were more physiologically/cognitively impaired as assessed by National Institute of Health Stroke Scale (NIHSS), with average scores of 16.8 (SD = 6.6), compared to 1.9 (SD = 2) for IHC (differences strongly significant between groups; *t* = −12.2; *p* < < 0.001). No adverse events were reported for any subjects as a result of TCD examination.

### Individual metric statistical comparisons

**Figure 3:**
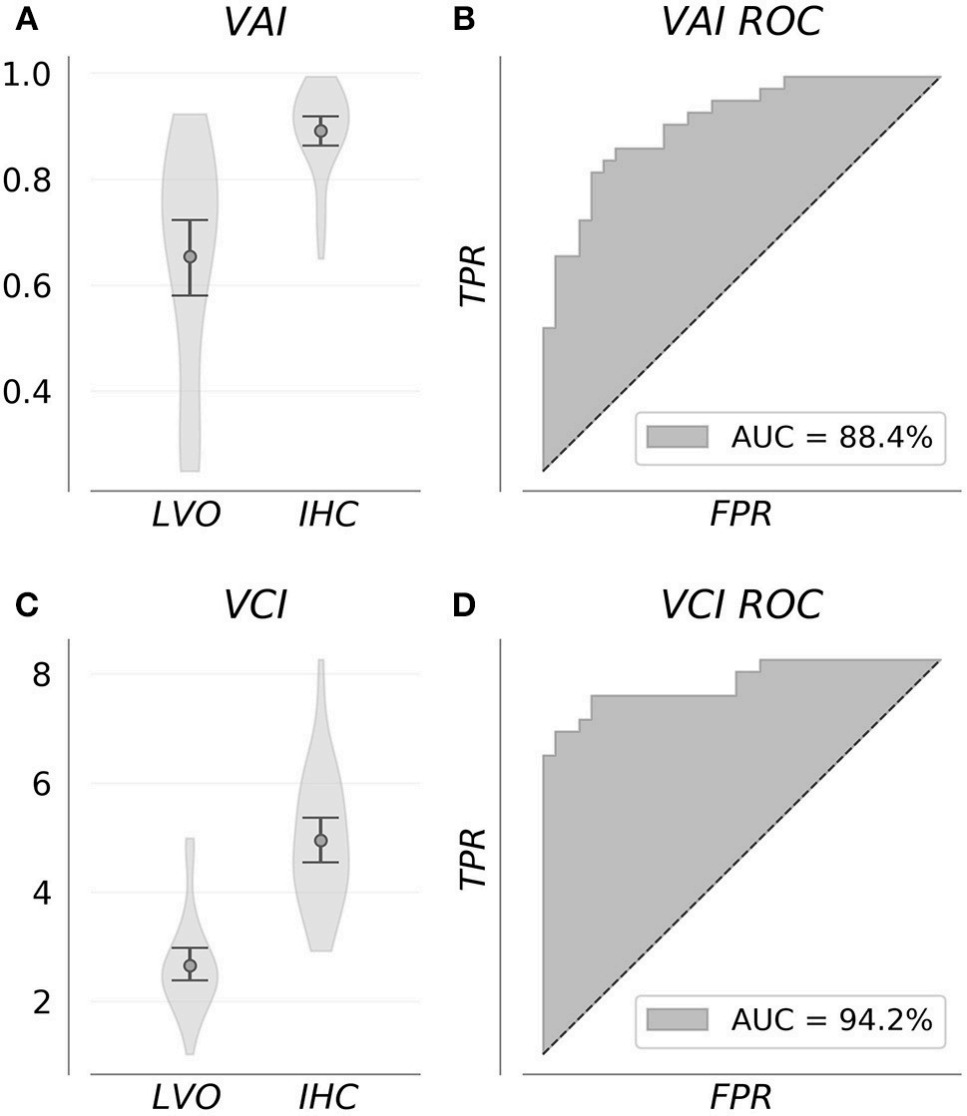
Group feature distributions **(A,C)** were significantly different for both metrics (*p* < < 0.001). Associated ROC curves **(B,D)** confirm both VAI and VCI provide diagnostically relevant information concerning the presence of LVO, with the greater separability observed for VCI suggesting it more information rich.

Figure 3 shows VAI and VCI metric distributions for LVO and IHC groups (A, C), and associated ROC curves depicting separability of group metrics (B, D). VAI means were greater for IHC subjects (0.89, CI = 0.86–0.92) relative to LVO (0.65, CI = 0.58–0.72). Associated ROC-AUC was observed at 88.4%, with significant group distribution differences confirmed by Mann-Whitney testing (*p* < < 0.001). Similarly, VCI means were greater for IHC subjects (4.95, CI = 4.55–5.36) relative to LVO (2.66, CI = 2.38–2.97); with associated ROC-AUC observed at 94.2%. Significant group distribution differences were likewise confirmed by Mann-Whitney testing (*p* < < 0.001). SEN/SPE/ACC at thresholds corresponding to Youden's maximal J are detailed in Table 1 for both metrics.

**Table 1:** Descriptive information and performance indicators comparing LVO and IHC groups for VAI and VCI metrics at thresholds corresponding to Youden's maximal J statistic.

**Metric**	**Mean (LVO, IHC)**	**CI (LVO, IHC)**	**AUC**	**SEN**	**SPE**	**ACC**
**LVO VS. IHC GROUP METRICS**						
*VAI*	0.65, 0.89	(0.58–0.72), (0.86–0.92)	0.88	0.82	0.82	0.82
*VCI*	2.66, 4.95	(2.38–2.97), (4.55–5.36)	0.94	0.91	0.88	0.89

*In column 3, CI refers to 95 percent Confidence Intervals around the respective means given in column 2*.

### Sensitivity analysis

**Figure 4:**
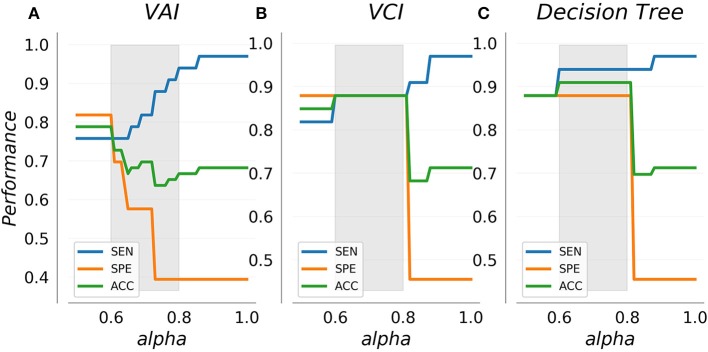
Cross-validated performance indicators for the VAI **(A)** and VCI **(B)** metrics as well as combined decision tree classifier **(C)**. Sensitivity increases with the alpha weighting parameter as specificity decreases. VAI specificity decreases rapidly with increased sensitivity, whereas VCI and the decision tree display a stable range (indicated in gray) wherein specificity and accuracy are optimal.

Figure 4 shows SEN, SPE, and ACC dependence on the alpha weighting parameter using leave-one-out cross-validation for each classifier. By definition, the sensitivity of each classifier increases with increased alpha. Interestingly, performance indicator trajectories vary substantially between the VAI classifier and the other two (VCI and decision tree). For VAI, observed SPE and ACC are optimal near the maximal J (alpha = 0.5), and rapidly degrade with increased prioritization of SEN corresponding to alpha greater than 0.6. In contrast, for VCI and the decision tree, a stable range of alpha exists away from the maximal J for which SPE, and ACC are optimized and all performance indicators are stable. This range, roughly 0.6–0.8, is indicated in gray in Figure 4. Above this range, SEN increases for VCI and decision tree are accompanied by precipitous decreases in ACC and SPE. Together these results suggest a natural optimal alpha range for prioritization of sensitivity for the VCI and decision tree classifiers.

**Figure 5:**
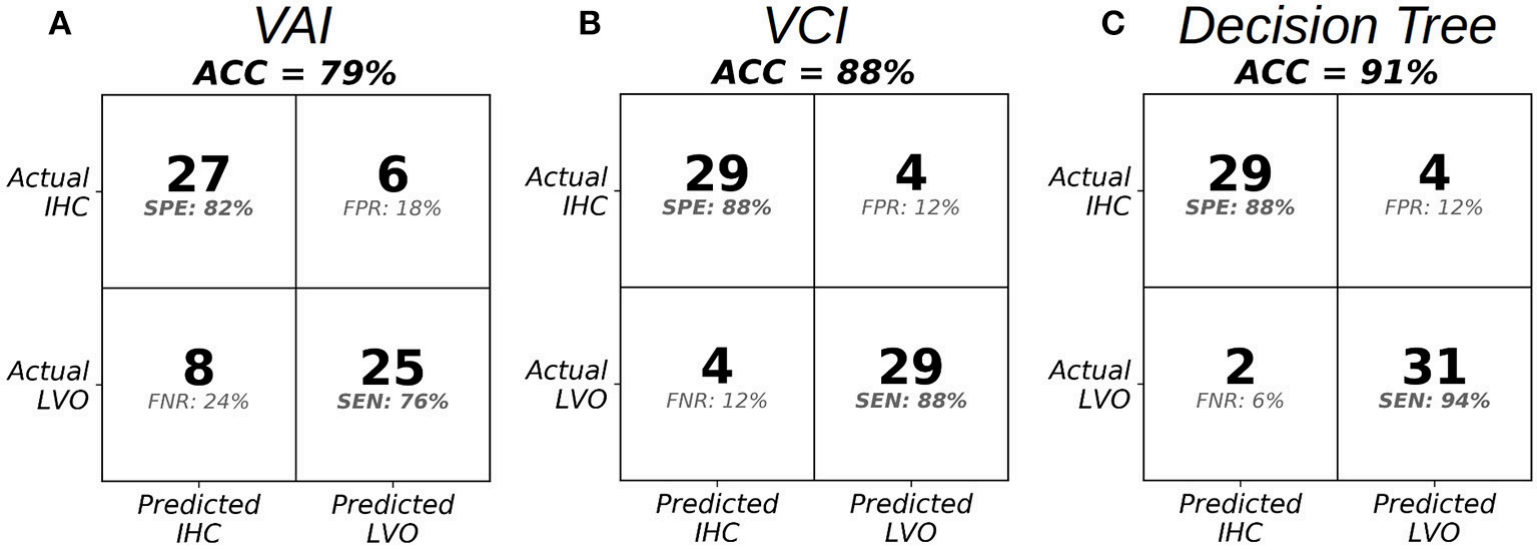
Confusion matrices at the specified threshold corresponding to alpha = 0.6 in the optimal range for VAI **(A)**, VCI **(B)**, and the Decision Tree Classifier **(C)**.

Figure 5 shows cross-validated confusion matrices for each classifier with alpha specified at 0.6, which represents the start of the optimal range for the VCI and decision tree classifiers, and the tail end of the optimal range for the VAI classifier. For VAI, we observed an overall accuracy of 79%, with SEN/SPE of 76%, and 82%, respectively. For VCI, we observed an overall accuracy of 88%, with SEN/SPE of 88%, and 88%, respectively. Finally, for the decision tree we observed an overall accuracy of 91%, with SEN/SPE of 94%, and 88%, respectively. Together, these results demonstrate the superiority of the VCI classifier relative to VAI. However, within the framework of the decision tree, VAI helped to increase SEN of LVO identification relative to VCI alone. Figure 5 results are summarized in Table 2.

**Table 2:** Performance indicators for leave-one-out cross-validated classifiers comparing LVO and IHC groups, with classification thresholds specified at alpha equal to 0.6.

**Classifier**	**SEN**	**SPE**	**ACC**
**CROSS-VALIDATED LVO CLASSIFIER PERFORMANCE METRICS**			
*VAI*	0.76	0.82	0.79
*VCI*	0.88	0.88	0.88
*Decision tree*	0.94	0.88	0.91

## Discussion

To our knowledge, this work represents the first published LVO decision criteria based on TCD variables which can be computed algorithmically and interpreted objectively. Results from all classifiers fall into the range observed in previous TCD studies using complex multi-vessel recording protocols (13, 17). Moreover, previous studies using predictive variables amenable to ROC analysis have not been subject to cross-validation in the manner we have performed here. Most importantly, these metrics substantially outperform stroke severity scales currently in prehospital use, which recent reviews suggest are unlikely to predict LVO with both high sensitivity and specificity (6, 7). Specifically, (6) published performance indicators for 5 such stroke assessment scales (RACE, 3ISS, LAMS, CPSSS, and PASS); reporting ACC and SEN capped at 74 and 64% across all scales. A sense of the potential for improvement upon these numbers can be gleaned from our simple decision tree which achieved cross-validated ACC and SEN exceeding 90%.

Previous work assessing TCD efficacy in detecting LVO align well with our current results. Tsivgoulis et al. (17) detected occlusions and stenoses based on the presence of the pathological waveforms described by Demchuk et al. (10) with SEN/SPE of 79 and 94%, respectively. A similar exam protocol was used by Brunser et al. (13), but with additional power M-mode criteria also considered, which achieved SEN/SPE of 90 and 94% detecting occlusion of any non-specific artery. The sensitivity and specificity observed for our metrics compare reasonably well with these previous results, which is especially encouraging considering our features were extracted from bilateral examination of a single vessel. Reliance on the MCA signals is pragmatic, but also represents a notable opportunity for improvement upon our current experimental paradigm. The MCA possesses the longest expected segment of probeable depths, and is thus most easily insonated and reliably located, but there is clearly more diagnostic information available in other vessels in the form of relative morphology and collateral flow which may improve performance in future experiments.

It is notable that the performance indicator curves we observed for VCI and the decision tree were extremely similar. This is a natural consequence of the decision criteria, which dictates that VAI is used only to decide “uncertain” subjects, thus serving mainly to improve upon VCI sensitivity to the degree allowable by the training data. Further work is needed to determine if there are specific occlusion types or patient demographics for which each metric works particularly better or worse, which should help to optimize decision criterion. In this configuration, VCI is doing the “heavy lifting” in our decision tree. It is an effective diagnostic metric because it is sensitive to the morphological structure of Demchuk's minimal, blunted, and dampened flows (10). The *blunted* waveform, for example, possesses an inherently smooth (i.e., low curvature) systolic complex, and is thus readily quantified by VCI. Forthcoming work will analyze in specific detail the manner in which VCI captures the subtle morphological variations associated with pathological LVO waveforms.

Some limitations of our study and directions for future work should be noted. The primary factor which could potentially inhibit generalisability of our results is the small sample size of our study. Much further data is required to refine estimates of morphological variability inherent in LVO and clinical control patient populations. Additionally, numerous important subgroup analyses are required to determine if/how TCD morphology depends on demographic and clinical factors (age, gender, occlusion type, etc). It should also be noted that the TCD technician's exposure to patient clinical information represents a potential source of bias which should be mitigated in future work by more thorough blinding. Finally, the relationships between curvature, heart rate, and stroke pathology require further investigation. In our sample, LVO subjects had an average heart rate of 87.9 (SD = 22.2) beats per minute (BPM), vs. 71.2 (SD = 11) BPM for IHC; which was significantly different between groups (*t* = 3.8, *p* < 0.001). However, there was no significant correlation between heart rate and curvature within either subject group (*r* = −0.006, *p* = 0.9 for IHC; *r* = −0.29, *p* = 0.1 for LVO). Moreover, when we use heart rate itself as a predictor to distinguish between groups, we observe an AUC of 73%, considerably underperforming both the VAI (88% AUC), and VCI (94% AUC) metrics. So, while it is possible that heart rate accounts for some degree of variance between groups, it remains unclear whether the effect is causal or correlative. It is certainly plausible that the lack of blood supply characteristic of LVO causes heart rate to increase; meaning heart rate is effectively part of the diagnostic signal. Further work is needed to establish how elevated heart rate might affect VCI when occlusion is not present.

One strength of the current approach is simplicity of data acquisition and communicability of decision criteria. However, as the amount of data we acquire increases, the subtle variations we will ultimately wish to detect will undoubtedly require more complicated and abstracted models. Given the efficacy of initial results, the road map to such models is encouraging. Incorporation of depth dependent and inter-hemispheric morphological dynamics across multiple vessels might ultimately allow precise prehospital localization of occlusion, and distinction between occlusive and hemorrhagic strokes. Moreover, digital rendering of individual subject vasculature, currently possible with emergent technologies such as 3D MRA time of flight imaging, could facilitate ultra rapid mapping and scanning across multiple vessels, as well as development of anatomically realistic mathematical models of cerebral hemodynamics. Such models could dramatically increase our understanding of the fluid mechanics involved in vascular occlusion, and the associated impact on morphological biomarkers like VCI.

## Conclusions

Our results suggest both VAI and VCI contain robust information concerning the presence of intracranial occlusion. Both are objective and real-time computable, and thus represent promising candidate metrics for the development of TCD-based prehospital LVO diagnostic systems. The feature distributions and classification performance indicators we observed suggest VCI may be superior to VAI for LVO detection, but even simple approaches to feature combination such as the decision tree analyzed herein may serve to further increase diagnostic accuracy. More data is needed to determine how well these decision criteria scale and generalize to a wider range of subject demographics and pathologies. Nonetheless, these results demonstrate the foundational potential for machine-learning approaches to TCD morphological analysis to enable faster and more widespread access to life saving medical intervention in the future.

## Ethics statement

This study was carried out in accordance with the recommendations of Declaration of Helsinki and The University of Tennessee College of Medicine Institutional Review Board with written informed consent from all subjects. All subjects gave written informed consent in accordance with the Declaration of Helsinki. The protocol was approved by the University of Tennessee College of Medicine Institutional Review Board (ID: 16-097).

## Author contributions

ST had access to all data used in the study and takes responsibility for data integrity and accuracy of data analysis. SW, RH, and TD study concept and design. ST and CT analysis. ST, CT, and NC interpretation of data. ST drafting of the manuscript. CT, NC, SW, RH, and TD critical revision of the manuscript for important intellectual content. ST statistical analysis. CT, NC, RH, SW, and TD technical or material support. RH and TD study supervision.

### Conflict of interest statement

At the time that this research was conducted, ST, CT, NC, SW, and RH were employees of Neural Analytics, Inc., and TD was a paid consultant. All authors either hold stock or stock options in Neural Analytics, Inc.
