# Supplementary material for: Decision Criteria for Large Vessel Occlusion Using Transcranial Doppler Waveform Morphology
Source: Front Neurol. 2018 Oct 17;9:847. doi: 10.3389/fneur.2018.00847 (PMC6199366; doi:10.3389/fneur.2018.00847)
Supplement: Supplementary file 1 [file Table_1.DOCX]

**Standards for Reporting of Diagnostic Accuracy (STARD) checklist.**

| **No.** | **Item** | **Section** |
| --- | --- | --- |
| **1** | Identification as a study of diagnostic accuracy using at least one measure of accuracy (such as sensitivity, specificity, predictive values, or AUC) | **Abstract** |
| **2** | Structured summary of study design, methods, results, and conclusions | **Abstract** |
| **3** | Scientific and clinical background, including the intended use and clinical role of the index test | **1.** |
| **4** | Study objectives and hypotheses | **1.** |
| **5** | Whether data collection was planned before the index test and reference standard were performed (prospective study) or after (retrospective study) | **Abstract, 1.** |
| **6** | Eligibility criteria | **2.1** |
| **7** | On what basis potentially eligible participants were identified (such as symptoms, results from previous tests, inclusion in registry) | **2.1** |
| **8** | Where and when potentially eligible participants were identified (setting, location and dates) | **2.1** |
| **9** | Whether participants formed a consecutive, random or convenience series | **2.1** |
| **10a** | Index test, in sufficient detail to allow replication | **2.2.2 – 2.2.4** |
| **10b** | Reference standard, in sufficient detail to allow replication | **2.1** |
| **11** | Rationale for choosing the reference standard (if alternatives exist) | **1.** |
| **12a** | Definition of and rationale for test positivity cut-offs or result categories of the index test, distinguishing pre-specified from exploratory | **2.4.1 – 2.4.3** |
| **12b** | Definition of and rationale for test positivity cut-offs or result categories of the reference standard, distinguishing pre-specified from exploratory | **2.1** |
| **13a** | Whether clinical information and reference standard results were available to the performers/readers of the index test | **2.1** |
| **13b** | Whether clinical information and index test results were available to the assessors of the reference standard | **2.1** |
| **14** | Methods for estimating or comparing measures of diagnostic accuracy | **2.3 – 2.4** |
| **15** | **How indeterminate index test or reference standard results were handled | **2.1** |
| **16** | **How missing data on the index test and reference standard were handled | **2.1** |
| **17** | Any analyses of variability in diagnostic accuracy, distinguishing pre-specified from exploratory | **2.4.2 – 2.4.3,**  **3.2 – 3.3** |
| **18** | **Intended sample size and how it was determined | **2.1** |
| **19** | Flow of participants, using a diagram | **3.1.**  **Diagram N/A** |
| **20** | Baseline demographic and clinical characteristics of participants | **3.1** |
| **21a** | Distribution of severity of disease in those with the target condition | **3.1** |
| **21b** | Distribution of alternative diagnoses in those without the target condition | **N/A** |
| **22** | Time interval and any clinical interventions between index test and reference standard | **3.1** |
| **23** | Cross tabulation of the index test results (or their distribution) by the results of the reference standard | **3.2, 3.3** |
| **24** | Estimates of diagnostic accuracy and their precision (such as 95% confidence intervals) | **3.2, 3.3** |
| **25** | Any adverse events from performing the index test or the reference standard | **3.1** |
| **26** | Study limitations, including sources of potential bias, statistical uncertainty, and generalisability | **4.** |
| **27** | Implications for practice, including the intended use and clinical role of the index test | **4.** |
| **28** | Registration number and name of registry | **N/A** |
| **29** | Where the full study protocol can be accessed | **N/A** |
| **30** | Sources of funding and other support; role of funders | **Funding Statement** |

**Appendix 1, Table 1.** The STARD (2015) list of essential items for reporting diagnostic accuracy are given, along with the section in the manuscript in which each criterion is satisfied. For further details on the STARD standards, see Bossuyt et al., (2015). STARD 2015: An updated list of essential items for reporting diagnostic accuracy studies. *Clinical Chemistry*, 61(12), 1446–1452. https://doi.org/10.1373/clinchem.2015.246280
